# Gender differences in the modifying effect of living arrangements on the association of sleep quality with cognitive function among community-dwelling older adults: a cross-sectional study

**DOI:** 10.3389/fpubh.2023.1142362

**Published:** 2023-05-05

**Authors:** Haifeng Yang, Lingzhong Xu, Wenzhe Qin, Fangfang Hu, Lei Li, Chenhui Chen, Wenlong Tang

**Affiliations:** ^1^Center for Health Management and Policy Research, School of Public Health, Cheeloo College of Medicine, Shandong University, Jinan, Shandong, China; ^2^NHC Key Lab of Health Economics and Policy Research, Shandong University, Jinan, Shandong, China; ^3^Center for Health Economics Experiment and Public Policy Research, Shandong University, Jinan, Shandong, China

**Keywords:** mild cognitive impairment, sleep quality, living arrangements, gender differences, older adults

## Abstract

**Background:**

Sleep quality is considered to be associated with cognitive function for older adults, but little is known about whether living with others can buffer mild cognitive impairment in older adults with poor sleep quality. The objective of this study was to examine the role of living arrangements in sleep quality and cognitive function among older adults aged 65 and over.

**Methods:**

2,859 older adults over 65 years old were selected by using multi-stage stratified sampling method. Cognitive function and sleep quality were measured using Mini-Mental State Examination (MMSE) and Pittsburgh Sleep Quality Index (PSQI). Binary logistic regression was performed to examine the relationship between sleep quality and mild cognitive impairment, and the interaction effects of sleep quality and living arrangements on mild cognitive impairment stratified by gender.

**Results:**

Poor sleep quality was associated with mild cognitive impairment among men and women regardless of living arrangements. The significantly protective role of living with others in reducing the incidence of mild cognitive impairment was found in men with poor sleep quality, but not in women.

**Conclusion:**

Targeted support for older adults with poor sleep quality may be effective in preventing mild cognitive impairment, and gender differences should be taken into account when promoting cohabitations.

## 1. Introduction

One of the most pressing concerns facing most nations is the aging of their populations, and China began to experience this trend at the close of the 20th century (1). Data from China’s seventh National Census showed that nearly 200 million people were aged over 65, accounting for 13.5% (2). It is estimated that by 2050, China will have 400 million people over 65, accounting for approximately 25% of the total population (3). There is no doubt that older individuals are often vulnerable to chronic physical and mental illness. With the aging of population, numerous serious health-related problems are in urgent need of attention (4). Dementia is one of the most common and serious diseases among older adults, which has been the top 10 leading causes of disability in low- and middle-income countries (5). China is now carrying a heavy burden of prevention and management due to having the most dementia sufferers in the world, accounting for about 25% of the global total (6). The early stages of dementia can be marked by some kinds of impairment in cognitive function, hence mild cognitive impairment (MCI) is an essential screening indicator and the best period of window to delay dementia for early recognition and intervention (7, 8).

MCI refers to impairment in one or more cognitive function domains, a state of possible transition between normal aging and dementia (7). MCI poses a serious threat to the quality of life among older adults, which may result in functional dependence and premature mortality (9). The prevalence of cognitive impairment rises along with the population’s aging trend and the rising life expectancy. Previously, it has been reported that the prevalence of cognitive impairment among Chinese community-dwelling older adults ranged from 10.2% (10) to 31.5% (11). One recent research showed a rate of cognitive impairment in the older adults over 65 even reached 54.9% (7), with a higher prevalence in women than men (12). The severe consequences and high prevalence of cognitive impairment will place enormous challenges to healthcare systems and hinder the advancement of successful and active ageing (13–15). In order to provide pertinent information for the prevention of dementia, it is important to investigate the associated risk and protective factors affecting cognitive impairment. Numerous studies have examined the causes of MCI in recent years, with an increasing focus on the relationship between sleep and cognitive function or dementia. A number of systematic reviews and meta-analyses have managed to ascertain whether the existing evidence supports the proposed risk contribution of sleep complaints to the developing process of cognitive impairment and dementia (16–19).

Sleep, a habitual behavior that takes a significant portion of lives, is crucial for preserving human health (20, 21). But sleep problem has increasingly become one of the main health issues affecting older people (22, 23). A national survey found that about 35% of Chinese older people reported poor sleep quality among 15,638 individuals (24). Meanwhile, higher odds of sleep problems among women than men in India, China, Russia, and South Africa were found according to data from the WHO Study on global aging and adult health survey (25). A growing body of research has linked low sleep quality to MCI. One study conducted among female older adults showed that those with sleep disorders of breathing were more likely to experience cognitive decline (26). Sleep disturbance is also prevalent and predictive of cognitive decline in older people (27). Following sleep disturbance or sleep loss, deficiencies in attention, learning and memory, emotional reactivity, and higher-order cognitive processes have all been observed (28). There have also been a number of clinical and physiological studies that have attempted to understand the mechanisms by which poor sleep quality is associated with cognitive impairment (16, 29).

However, few studies have examined the role of environmental conditions, such as living arrangements, in the relationship between sleep quality and cognitive function in older adults. Living arrangements matter to the health and well-being of the older adults, as the home is a significant factor of establishing social roles and providing older persons with social support and contact (30). The incidence of living alone is higher among older persons, despite the fact that it has increased for younger and middle-aged adults (31). With more than 50% of China’s old population living alone, living arrangements have become a social concern that cannot be disregarded (32). Some studies supported that living alone was associated with poor health and mental consequences such as decreased physical and cognitive function, loneliness, and suicidal ideation among older adults (33–36). What’s more, the degree to which living arrangements impact health varies across men and women (37–39). The stress-buffering model indicated that the moderator can reduce the likelihood of adverse health outcomes by buffering stress before it has an effect on health outcomes (40). Therefore, we speculate that living with others, generating a stable and intimate companionship as support resources, may buffer the stress from sleep disorders to reduce the impairment of cognitive function.

In light of the disparities between men and women in MCI, sleep and the health effects of living arrangements, it would be wise to consider gender differences when probing into the associations between them. Previous research has shown that there existed gender differences in the relationship between sleep disorders and MCI since sex hormones play a key role in regulating these conditions (41). Besides, the association between living alone and cognitive impairment seems to be distinctly different among males and females. Evidence from longitudinal study suggested that men who lived alone were at a higher risk of cognitive impairment whereas there is not such trend among women (39).

To our knowledge, no study has examined the buffering effect of living with others in the relationship between sleep quality and cognitive function. Therefore, the aims of this study were as follows: (1) to analyze the relationship between poor sleep quality and MCI among older adults, (2) to investigate whether there is a buffering effect of living with others on the relationship between poor sleep quality and MCI among older adults, and (3) to explore whether gender influences such effect.

## 2. Methods

### 2.1. Data and sample

Data were gathered from the 2020 Household Health Interview Survey, which sought to investigate inhabitants’ health state, demand for, and use of, healthcare services. This cross-sectional survey was performed in Taian City, Shandong Province, China. Participants were chosen using stratified multi-stage random sampling from each of the six administrative districts (4 counties and 2 districts). Three or four sub-districts or towns were randomly chosen from each district or county in Taian City in the first stage using the probability proportionate to size sampling method (PPS), based on the level of socioeconomic development and geographic location, for a total of 20 sub-districts or towns. Eight villages and eight residency committees were chosen independently (PPS) from each town and subdistrict in the second stage, for a total of villages and committees. Lastly, an average of 50 families was randomly chosen from each village or residency committee (simple random sampling). 7,920 out of a total of 7,945 selected homes completed the entire survey. Finally, 2,859 older persons aged 65 and over who had not been diagnosed with dementia by doctors made up the research sample out of the 8,542 participants in the survey. A paper-based questionnaire was used to administer to every participant face-to-face by trained interviewers in their homes.

### 2.2. Measures

#### 2.2.1. Mini-mental state examination

Cognitive function was assessed by Mini-Mental State Examination. A total of 30 items evaluates orientation (0–10), immediate recall (0–3), delayed recall (0–3), attention or concentration capacity (0–5), and language ability (0–9) (42). Given that cognitive function and educational attainment are correlated, the cut-off value for MCI was adjusted to account for this relationship; it was set at ≤17 for illiteracy, ≤20 for primary school, and ≤ 24 for junior high school and above (43, 44).

#### 2.2.2. Pittsburgh sleep quality index

Sleep quality was measured by using Pittsburgh Sleep Quality Index (PSQI) (45). 19 items and 7 dimensions consist of the whole scale, which measures subjective sleep quality, sleep latency, sleep duration, habitual sleep efficiency, sleep disturbances, usage of sleep medication, and daytime dysfunction. Each subdimension drops score into 0, 1, 2, and 3 based on the entries it contains, the total score of the scale is the sum of the seven dimensions, with a higher score indicating the severe sleep problem. Participants with a score of >5 were considered to be with poor sleep quality (46), and correspondingly, those who scored ≤5 were thought to be with good sleep.

#### 2.2.3. Living arrangements

Living arrangements were collected by asking “How many persons have lived in your home in the past 6 months,” and the answer “only me” was defined as living alone.

#### 2.2.4. Covariates

We included as covariates confounding factors that may influence cognitive function according to previous studies. Sociodemographic characteristics included gender (male/female), age, residence (village/city or town), education (illiteracy or low literacy/primary school/junior high school and above), marital status (with spouse/ without spouse) (47). Life behaviors including, smoking (yes/no), drinking (yes/no), taking exercise (yes/no), and drinking tea (yes/no) were collected (48, 49). In addition, we also collected some health-related variables. By conducting physical measurement, body mass index (BMI) was calculated as weight (kg) by the square of height (m^2^) (50). The number of chronic diseases was categorized to 3 statuses: 0 represented no chronic disease, 1 represented single chronic disease, and ≥2 represented multimorbidity (50). Additionally, it was demonstrated that cognitive performance was related to hearing and vision difficulty (51). Self-perceived difficulty in hearing was obtained by asking participants to “in the past 6 months, whether (1) you hardly heard clearly, (2) or needed others to raise their voice, or (3) hear clearly,” answer (1) and (2) were classified as difficulty in hearing. Self-perceived difficulty in vision was obtained by asking “How difficult it was for you to recognize an acquaintance 20 m away within the past 6 months,” and the person who reported difficulty was considered as a case of vision impairment.

### 2.3. Statistical analysis

IBM SPSS statistical software (version 26.0) was used to conduct all statistical analyses. Firstly, classified variables were provided as frequency and percentage, and continuous variables (age and BMI) were presented as mean and standard deviation (SD). Differences in MCI among older adults in sociodemographic characteristics, life behaviors, and health-related variables were analyzed using chi-square test of independence and two independent-sample *t*-test according to gender. Secondly, the associations between poor sleep quality and cognitive impairment in men and women were analyzed by using logistic regression model on the basis of gradually adjusting the control variables. Thirdly, according to the status of sleep quality and living arrangements, the participants were stratified into four groups (see details in Tables 1, 2) to investigate the interaction effects of sleep quality and living arrangements on MCI by using binary logistic regression. Statistical significance was defined as a two-tailed *p*-value less than 0.05.

**Table 1:** Interaction effects of sleep quality and living arrangement on MCI in men.

	Model 4	Model 5	Model 6
	OR (95% CI)	OR (95% CI)	OR (95% CI)
Sleep quality + Living arrangements (ref: Good sleep quality + Living with others)			
Poor sleep quality + Living with others	1.591 (1.172, 2.161)**	1.567 (1.151, 2.133)**	1.499 (1.092, 2.057)*
Good sleep quality + Living alone	1.312 (0.671, 2.569)	1.286 (0.653, 2.529)	1.191 (0.600, 2.362)
Poor sleep quality + Living alone	2.640 (1.218, 5.723)*	2.640 (1.211, 5.750)*	2.519 (1.138, 5.576)*
Age	1.068 (1.040, 1.097) ***	1.069 (1.040, 1.099) ***	1.053 (1.023, 1.083) ***
Residence (ref: Village)			
City or town	0.705 (0.516, 0.964)*	0.714 (0.520, 0.979)*	0.820 (0.593, 1.124)
Education (ref: Illiteracy or low literacy)			
Primary school	0.688 (0.459, 1.031)	0.709 (0.472, 1.066)	0.742 (0.490, 1.123)
Junior high school and above	1.912 (0.836, 1.701)	1.252 (0.873, 1.795)	1.441 (0.994, 1.087)
Marital status (ref: With spouse)			
Without spouse	1.059 (0.611, 1.837)	1.048 (0.601, 1.827)	1.067 (0.608, 1.874)
Smoking (ref: No)			
Yes		1.039 (0.756, 1.427)	0.978 (0.707, 1.353)
Drinking (ref: No)			
Yes		0.861 (0.639, 1.159)	0.850 (0.627, 1.153)
Having tea (ref: No)			
Yes		0.610 (0.452, 0.824)**	0.612 (0.450, 0.831)**
Taking exercise (ref: No)			
Yes		0.737 (0.314, 1.731)	0.658 (0.276, 1.570)
BMI			0.955 (0.913, 0.999)*
Number of chronic diseases (ref: 0)			
1			0.964 (0.665, 1.396)
≥2			0.742 (0.512, 1.074)
Self-perceived difficulty in hearing (ref: No)			
Yes			2.166 (1.599, 2.935)***
Self-perceived difficulty in vision (ref: No)			
Yes			1.154 (0.854, 1.559)

BMI, body mass index; OR, odds ratio; CI, confidence interval.

*p < 0.05, **p < 0.01, ***p < 0.001.

**Table 2:** Interaction effects of sleep quality and living arrangement on MCI in women.

	Model 7	Model 8	Model 9
	OR (95% CI)	OR (95% CI)	OR (95% CI)
Sleep quality + Living arrangements (ref: Good sleep quality + living with others)			
Poor sleep quality + living with others	1.489 (1.155, 1.918)**	1.474 (1.143, 1.901)*	1.362 (1.048, 1.770)*
Good sleep quality + living alone	0.911 (0.583, 1.423)	0.896 (0.571, 1.405)	0.906 (0.572, 1.434)
Poor sleep quality + living alone	1.443 (0.948, 2.197)	1.420 (0.931, 2.168)	1.332 (0.861, 2.062)
Age	1.085 (1.061, 1.111)***	1.087 (1.062, 1.112)***	1.074 (1.048, 1.100)***
Residence (ref: Village)			
City or town	0.558 (0.442, 0.704)***	0.574 (0.454, 0.726)***	0.582 (0.457, 0.742)***
Education (ref: Illiteracy or low literacy)			
Primary school	0.437 (0.317, 0.604)***	0.442 (0.320, 0.611)***	0.449 (0.333, 0.625)***
Junior high school and above	0.856 (0.600, 1.221)	0.869 (0.609, 1.241)	0.975 (0.676, 1.405)
Marital status (ref: With spouse)			
Without spouse	1.156 (0.846, 1.578)	1.152 (0.842, 1.576)	1.070 (0.775, 1.478)
Smoking (ref: No)			
Yes		1.764 (0.794, 3.918)	1.745 (0.767, 3.969)
Drinking (ref: No)			
Yes		1.059 (0.622, 1.805)	1.010 (0.585, 1.745)
Having tea (ref: No)			
Yes		0.792 (0.632, 0.993)*	0.867 (0.686, 1.096)
Taking exercise (ref: No)			
Yes		0.563 (0.322, 0.983) *	0.524 (0.296, 0.928) *
BMI			0.986 (0.945, 1.018)
Number of chronic diseases (ref: 0)			
1			1.004 (0.736, 1.371)
≥2			1.111 (0.826, 1.495)
Self-perceived difficulty in hearing (ref: No)			
Yes			2.427 (1.912, 3.082) ***
Self-perceived difficulty in vision (ref: No)			
Yes			1.239 (0.970, 1.581)

BMI, body mass index; OR, odds ratio; CI, confidence interval.

*p < 0.05, **p < 0.01, ***p < 0.001.

## 3. Results

### 3.1. Sample description

Table 3 shows descriptive statistics for the participants. Among 2,859 older adults, 41.4% were men and 58.6% were women. The rates of MCI were 22.9 and 29.7% in men and women, respectively. Compared with participants without MCI, both men and women with MCI were more likely to be older, living in small town, uneducated, without spouse, have a lower intake of tea, with slightly lower BMI value, and have difficulty in hearing and vision, poor sleep quality and live alone. In addition, the proportion of women with MCI of smokers was significantly higher than non-smokers.

**Table 3:** Characteristics of the older adults aged 65 and over.

Variables	Total (*n* = 2,859)	Men (*n* = 1,184)			Women (*n* = 1,675)		
		Mild cognitive impairment			Mild cognitive impairment		
		No^a^ (*n* = 913)	Yes^b^ (*n* = 271)	*p*	No^a^ (*n* = 1,178)	Yes^b^ (*n* = 497)	*p*
Age, mean ± SD	71.29 ± 5.04	70.77 ± 4.719	72.45 ± 5.95	<0.001^c^	70.72 ± 4.54	72.95 ± 5.75	<0.001^c^
Residence				0.049^d^			<0.001^d^
Village	1,718 (60.1)	588 (75.4)	192 (24.6)		607 (64.7)	331 (35.3)	
City or town	1,141 (39.9)	325 (80.4)	79 (19.6)		571 (77.5)	166 (22.5)	
Education				0.031^d^			<0.001^d^
Illiteracy or low literacy	1,393 (48.7)	197 (74.1)	69 (25.9)		737 (65.4)	390 (34.6)	
Primary school	666 (23.3)	272 (82.2)	59 (17.8)		280 (83.6)	55 (16.4)	
Junior high school and above	800 (28.0)	444 (75.6)	143 (24.4)		161 (75.6)	52 (24.4)	
Marital status				0.010^d^			<0.001^d^
With spouse	2,089 (73.1)	786 (78.4)	216 (21.6)		800 (73.6)	287 (26.4)	
Without spouse	770 (26.9)	127 (69.8)	55 (30.2)		378 (64.3)	210 (35.7)	
Smoking				0.452^d^			0.011^d^
Yes	408 (14.3)	295 (78.5)	81 (21.5)		16 (50.0)	16 (50.0)	
No	2,451 (85.7)	618 (76.5)	190 (23.5)		1,162 (70.7)	481 (29.3)	
Drinking				0.086^d^			0.406^d^
Yes	864 (30.2)	614 (78.6)	167 (21.4)		55 (66.3)	28 (33.7)	
No	1,995 (69.8)	299 (74.2)	104 (25.8)		1,123 (70.5)	469 (29.5)	
Having tea				0.001^d^			0.021^d^
Yes	1,906 (66.7)	684 (79.7)	174 (20.3)		758 (72.3)	290 (27.7)	
No	953 (33.3)	229 (70.2)	97 (29.8)		420 (67.0)	207 (33.0)	
Taking exercise							
Yes	88 (3.1)	21 (72.4)	8 (27.6)	0.542^d^	35 (59.3)	24 (40.7)	0.060^d^
No	2,771 (96.9)	892 (77.2)	263 (22.8)		1,143 (70.7)	473 (29.3)	
BMI, mean ± SD	24.34 ± 3.49	24.08 ± 3.31	23.43 ± 3.24	0.002^c^	24.83 ± 3.50	24.12 ± 3.78	<0.001^c^
Number of chronic diseases				0.402^d^			0.092^d^
0	662 (23.2)	212 (76.0)	67 (24.0)		280 (73.1)	103 (26.9)	
1	999 (34.9)	319 (75.6)	103 (24.4)		415 (71.9)	162 (28.1)	
≥2	1,198 (41.9)	382 (79.1)	101 (20.9)		483 (67.6)	232 (32.4)	
Self-perceived difficulty in hearing				<0.001^d^			<0.001^d^
Yes	1,063 (37.2)	345 (68.3)	160 (31.7)		304 (54.5)	254 (45.5)	
No	1796 (62.8)	568 (83.7)	111 (16.3)		874 (78.2)	243 (21.8)	
Self-perceived difficulty in vision				0.002^d^			<0.001^d^
Yes	1,641 (57.4)	471 (73.6)	169 (26.4)		663 (66.2)	338 (33.8)	
No	1,218 (42.6)	442 (81.2)	102 (18.8)		515 (76.4)	159 (23.6)	
Living arrangements				0.012^d^			0.004^d^
Alone	513 (17.9)	97 (68.8)	44 (31.2)		239 (64.2)	133 (35.8)	
With others	2,346 (82.1)	816 (78.2)	227 (21.8)		939 (72.1)	364 (27.9)	
Poor sleep quality				<0.001^d^			<0.001^d^
Yes	914 (68.0)	290 (71.1)	118 (28.9)		563 (65.9)	291 (34.1)	
No	1,945 (32.0)	623 (80.3)	153 (19.7)		615 (74.9)	206 (25.1)	

SD, standard deviation; BMI, body mass index.

a Without MCI.

b With MCI.

c *t*-test.

d Chi-square test.

### 3.2. Association between sleep quality and MCI

Table 4 shows the results of binary logistic regression for association between sleep quality and MCI among older adults. In model 1, poor sleep quality was a risk factor for MCI among both men (OR: 1.640, 95% CI: 1.234–2.712) and women (OR: 1.510, 95% CI: 1.211–1.884). After controlling for sociodemographic, behavioral, and health-related variables, the effect still existed in men (OR: 1.565, 95%CI: 1.168–2.097) and women (OR:1.387, 95%CI: 1.168–2.097).

**Table 4:** Binary logistic regression for association between sleep quality and MCI.

	Men	Women
	AOR (95% CI)	AOR (95% CI)
Model1		
Good sleep quality	Ref	Ref
Poor sleep quality	1.640 (1.234, 2.172) **	1.510 (1.211, 1.884) ***
Model 2		
Good sleep quality	Ref	Ref
Poor sleep quality	1.628 (1.224, 2.166)**	1.500 (1.201, 1.873)***
Model 3		
Good sleep quality	Ref	Ref
Poor sleep quality	1.565 (1.168, 2.097)**	1.387 (1.168, 2.097)**

Model 1: adjusted for age, residence, education and marital status.

Model 2: adjusted for age, residence, education, marital status, smoking, drinking, having tea, taking exercising, and living arrangements.

Model 3: further adjusted for BMI, number of chronic disease, self-perceived hearing difficulty, self-perceived vision difficulty.

AOR, adjusted odds ratio; CI, confidence interval.

*p < 0.05, **p < 0.01, ***p < 0.001.

### 3.3. Interaction effects analyses

Table 1 summarizes the combined effects of sleep quality and living arrangements on MCI in men. In Model 4, men with poor sleep quality who lived with others (OR = 1.591, 95% CI: 1.172, 2.161) were more likely to have MCI in comparison with those with good sleep who lived with others, and so did those with poor sleep quality who lived alone (OR = 2.640, 95% CI: 1.218–5.723). Model 5 shows similar results with approximate OR values. After further controlling for health-related variables (Model 6), the estimates aforementioned were attenuated but remained significant despite the significant effects of BMI and hearing difficulties on MCI. Men with poor sleep quality who lived with others (OR = 1.499, 95% CI: 1.092–2.057) as well as those with poor sleep quality who lived alone (OR = 2.519, 95% CI: 1.138–5.576) were both more likely to have MCI in comparison with those with good sleep who lived with others. Table 2 shows that women with poor sleep quality who lived with others had a significantly higher risk of MCI (OR = 1.489, 95% CI: 1.155–1.918) in Model 7. In the fully adjusted model (Model 9), the effect was diminished but still significant (OR = 1.362, 95% CI: 1.048–1.770). The results highlight the important buffering role of living with others on MCI in males with poor sleep quality, but not in females.

## 4. Discussion

This study investigated the relationship between sleep quality and cognitive function among male and female older adults aged 65 and over, and then further considered the interaction effect of poor sleep quality and living arrangements on MCI. Our main findings can be summarized as follows: (1) both men and women who had poor sleep quality were more likely to suffer from MCI and (2) the significantly protective role of living with others in reducing the incidence of MCI was found in men with poor sleep quality, but not in women.

In this study, the overall rate of cognitive impairment among older adults aged over 65 was 23.4%, which was in line with the prevalence of one study conducted among Chinese community-dwelling older adults (52). Besides, consistent with the literature, women reported a higher rate of MCI than men (8, 12). One possible explanation can be that women have a longer life expectancy than men (53), and cognitive impairment is associated with age-related neurodegenerative diseases (50), the incidence of cognitive impairment increases as they age. The findings further indicated that the prevention of cognitive impairment should not ignore gender differences.

After controlling sociodemographic characteristics, life behaviors and health-related variables, male and female older adults with poor sleep quality tend to experience higher risks of MCI. This result supports the viewpoint from previous observations that older adults with sleep problems were more likely to report severer subjective cognitive decline and poor cognitive performance in longitudinal and cross-sectional studies (54, 55). As one of the sleep problems, sleep-disordered breathing has been shown to cause early potentially reversible alterations in Alzheimer’s disease biomarkers (56), which may predict individual risk to develop Alzheimer’s disease (18). A network of state-modulating neurons in the hypothalamus and brain stem participates in the dynamic process of sleep, which interacts with the circadian and homeostatic systems to produce stable sleep and awake cycles every day (57). Due to the lack of continuous sleep or sleep duration, they may need to nap during the daytime, and this lack of wakefulness can cause problems with cognitive function including domains of memory, orientation, or concentration (58).

We observed that living with others had a buffering effect on cognitive impairment in male participants with sleep problems. There is a wide range of supporting ideas or evidence that living with others can directly or indirectly improve cognitive performance in older adults. To begin with, in traditional Chinese norms, it is a symbol of happiness for older adults to live with their spouse or children to enjoy the joys of family life (39). Older adults who lived with a spouse had much better objectively measured physical functions due to better support and motivation for exercise (50). It can be reasonably speculated that older adults are more likely to go for walks or visit friends and relatives by following their co-residents like spouse or children. This gentle form of walking has been suggested to be associated with the maintenance of cognitive function (51). In terms of the supportive environment created by living with others, the benefits of emotional support on older adults’ cognitive function do matter, as well. Generally, living with other people can benefit cognitive reserve in older adults by expanding their social networks and creating more stable social bonds through family ties (59). The positive emotions that older adults experience from high perceived social support are predictors of good cognitive function (52).

In the present study, our finding is partially consistent with previous longitudinal research, which found that living alone only negatively affected cognitive function in men (30). Males may experience limitations as a result of worries about the physical or emotional well-being of others, whether they live with spouse or children and grandchildren (60). Therefore, men are more likely to avoid or abstain from unhealthy habits such as smoking and drinking that have been shown to be risk factors of cognitive decline (61). Besides, men often rely on their partners more often than women as their main source of emotional and social support for confidence (62). What’s more, living with others is itself a predictor of better sleep quality (63). Hence, men are more likely to gain a better sense of well-being, physical activity motivation, and emotional support, thereby obtaining a lower risk of cognitive impairment. On the contrary, no cushioning effect of cohabitation on MCI was found in women. This may result from that women often take on prominent dedication tasks in family, taking care of not only their spouse but also their children and grandchildren (63). In this case, women who have sleep complaints are still burdened with trivia rather than seeking an outlet for relaxation or relief. As has been proposed by Jeon, living alone seems to be advantageous to women (64).

Conversely, the state of empty-nesters is easily associated with poorer physical, mental, and social interactions in men (65). As we mentioned earlier, older adults with sleep problems are at a cognitive disadvantage, and living alone can exacerbate this vulnerability with negative effects on physical, psychological, and social interactions. In a comparative study, male adults aged 70 and over who lived alone in the community were associated with lower physical function scores than those living with a spouse or partner (66), which may predict poor cognitive performance (67). Emotionally, living alone has been proven to be associated with severe depression and wide psychological distress (68, 69). As we know, psychological risk factors influence people’s cognitive trajectories, depression, anxiety, and decreased psychological resilience are somewhat contributors to cognitive decline (70, 71). Additionally, people who live alone were more likely to feel more socially isolated and had fewer social networks, both of which have been linked to cognitive decline (72, 73). And men were more prone than women to feel disconnected from society when they lived alone (74). Therefore, paying attention to the dual effects of sleep problems and living alone in men is a key point in preventing cognitive decline. But for women, we should not stereotype that living alone, loneliness, and isolation are always interrelated and simultaneous, or just conflate them (75). Living alone does not mean less social engagement and decreased psychological well-being, especially among older female adults (69). Though they have difficulty sleeping a comfortable and intact night, they can arrange more time to participate in physical activities and intellectually stimulating activities like square dancing or playing cards with friends to maintain better cognitive performance (76–78).

The findings on living with others being buffer of association between poor sleep quality and MCI might have essential implications for older adults. It is suggested that health managers like family doctors have the responsibility to understand and improve sleep quality in older people to promote their cognitive reserve. In addition, it is advocated for adult children to live with their older parents in order to create a more supportive environment with a caring, emotional, and behavioral assistant. Moreover, to better target MCI prevention, more gender-specific measures should be proposed on the basis of mastering health-related information including the physical, psychological, and social state.

There are several limitations that need to be stated here in this study. Firstly, given the cross-sectional design of the present study, we were unable to determine the causal association between sleep quality and cognitive function. Secondly, we have some concerns about using PSQI to measure sleep quality, as it only measures sleep in the month preceding the evaluation and lacks sufficient information about sleep breathing disorders (although including snoring) which may limit the comprehensive assessment of sleep quality and its relationship with MCI; meanwhile, the lack of assessment of mood may lead to insufficient inclusion of control variables. Thirdly, the population in this study was merely from Taian, Shandong Province, which is not conducive to generalizing the conclusion to the wider population of older adults, a representative sample from more geographical areas will be needed in the future. Finally, information about living arrangements was collected only as living alone and not alone, it is crucial to understand whom the older adults are living with to analyze its effect on MCI in more detail in the future.

## 5. Conclusion

Our results indicate that MCI was positively associated with poor sleep quality among older adults. The research further demonstrates the protective role of living with others on the relationship between sleep quality and MCI in only men. For men with poor sleep quality, living with others creates a more supportive environment that buffers the negative impact of poor sleep quality on cognitive function. These results suggest that more support and intervention strategies for cognitive function improvement should be provided for older adults with poor sleep quality, and gender differences should be taken into account when promoting cohabitations.

## Data availability statement

The raw data supporting the conclusions of this article will be made available by the authors, without undue reservation.

## Ethics statement

The studies involving human participants were reviewed and approved by the Ethical Committee of the Centre for Health Management and Policy Research, Shandong University (approval number: LL20191220). The patients/participants provided their written informed consent to participate in this study.

## Author contributions

HY designed the study, reviewed the literature, and wrote the manuscripts. LX provided the datasets and revised the draft. WQ refined the ideas and interpreted the results. FH contributed to data collection. LL, CC, and WT contributed to statistical analysis. All authors contributed to the article and approved the submitted version.

## Funding

This work was supported by the National Natural Science Foundation of China (71974118 and 72204145).

## Conflict of interest

The authors declare that the research was conducted in the absence of any commercial or financial relationships that could be construed as a potential conflict of interest.

## Publisher’s note

All claims expressed in this article are solely those of the authors and do not necessarily represent those of their affiliated organizations, or those of the publisher, the editors and the reviewers. Any product that may be evaluated in this article, or claim that may be made by its manufacturer, is not guaranteed or endorsed by the publisher.
